# Understanding Violence Toward Canadian Nurses From Patients’ Family and Visitors as a Potentially Psychologically Traumatic Event

**DOI:** 10.1155/jonm/3920566

**Published:** 2026-09-29

**Authors:** Kim Ritchie, Rosemary Ricciardelli, Matthew S. Johnston, Andrea D’Alessandro, Yuanxin Xue, Andrea M. Stelnicki, Margaret McKinnon, R. Nicholas Carleton

**Affiliations:** ^1^ Department of Psychiatry and Behavioral Neurosciences McMaster University Hamilton Canada mcmaster.ca; ^2^ Fisheries and Marine Institute Memorial University of Newfoundland St. John’s Canada mun.ca; ^3^ Department of Social Science University of New Brunswick (Saint John) Saint John Canada; ^4^ Temerty Faculty of Medicine University of Toronto Toronto Canada utoronto.ca; ^5^ High Point Psychology Calgary Canada; ^6^ Department of Psychology University of Regina Regina Canada uregina.ca

**Keywords:** family, nurse retention, psychological trauma, visitors, workplace violence

## Abstract

**Aim:**

In this study, we explore qualitatively the types and extent of violence perpetrated against Canadian nurses.

**Background:**

With the growing shortage of professional nurses globally, prioritizing the safety and well‐being of nurses is essential. Workplace violence is a critical factor that may aggravate exposure to potentially psychologically traumatic events (PPTEs) and negatively affect nurses’ mental health and well‐being. Research has found that patients’ family members and other visitors are the second highest perpetrators of health care violence against nurses.

**Method:**

Using a grounded theory‐informed approach to thematic analysis, we engaged in a secondary qualitative analysis of open‐text survey responses from a national survey of Canadian nurses’ (*n = *168) experiences of violence conducted between May and September 2019.

**Results:**

Nurses described their experience of violence from patients’ family and other visitors as the most impactful PPTE they experienced at work in open‐ended text box responses. Four primary themes were produced, each characterizing nurses’ experiences of visitor violence: Normalization of harm/violence, verbal abuse/harassment, physical violence, and emotional abuse.

**Conclusions:**

We put forth proactive strategies to enhance organizational and leadership response and reporting structures to improve the safety and mental well‐being of Canadian nurses.

**Implications for Nursing Management:**

Priority actions for nursing leaders, organizations, and managers could include strengthening visitor management protocols, improving security escalation pathways, simplifying reporting systems, reinforcing just culture, and providing postincident support and updated training for nurses.

## 1. Introduction

Canadian nurses appear to be at a disproportionately higher risk than the general public for exposures to potentially psychologically traumatic events (PPTEs) [1] and other diverse mental health challenges [1, 2]. PPTEs are one way to describe how someone may become indirectly (e.g., vicarious and witnessing) or directly (e.g., happens to them) exposed to violence, sexual violence, serious injury, severe human suffering, life‐threatening illness, serious accidents, threats, and/or death of another person [3]. In a recent study, Canadian nurses reported frequent exposure to PPTEs in the course of their work, and when participants were asked about their worst or most distressing event, physical assault (12.2%) was identified as the most prevalent event [1].

As an occupational group, nurses are known to be at high risk for exposure to workplace violence from patients, patients’ families, and colleagues, even relative to other health care professionals [4, 5]. Violence toward nurses can take on many forms, including threats, verbal and emotional abuse, physical assault, unwanted sexual comments or advances, and harassment [6]. The pervasive nature and extent of violence against nursing staff has led to the widely accepted and disturbing notion that violence is part and parcel of the nursing occupation [4, 6, 7]. The root causes of violence against health care workers could include staff shortages (which were exacerbated by the COVID‐19 pandemic) that lead to patient and family frustration with care, worker exploitation spawned by gender, racial, and migration status discrimination, as well as inadequate working conditions [8, 9].

Despite widespread recognition of workplace violence as a major issue in nursing, critical gaps persist in organizational and leadership responses. A systematic review of workplace violence in health care found human resource management often fails to implement effective strategies to mitigate violence and address its impacts on staff mental health [10]. In many institutions, reporting systems remain underutilized because of overly complicated procedures, lack of managerial follow‐up, and cultural normalization of violence [11]. Elsharkawy et al. [12] note, even when prevention policies exist, enforcement is uneven and punitive sanctions against perpetrators are rarely applied. Consequently, many nurses perceive that leadership tolerates or minimizes violence, which undermines trust, discourages reporting, and perpetuates a culture where violence is normalized. Understanding these gaps is critical to inform policy, practice, and safer work environments.

To underscore the severity of the problem in Canada (i.e., for context, not in relation to the current study’s exposure window), the Canadian Federation of Nurses Unions (CFNU) national report, Enough is Enough: Putting a Stop to Violence in the Health Care Sector, found “the majority believe patient safety is declining, or not improving, and workplace violence in health care is a serious and growing problem” (CFNU) [13]. The federal Standing Committee on Health’s report, Violence Facing Health Care Workers in Canada, similarly identified workplace violence as a critical concern and made several recommendations to support the dissemination and scaling‐up of best practices to prevent workplace violence in health care settings, including “education and training of health care professionals in the de‐escalation of violence; undertaking violence risk assessments of health care organizations, departments, and individual patients; flagging of patients who pose a risk for violent behavior; personal safety response systems that allow health care providers to call for help; and security personnel and protocols to respond to violence” [14]. Subsequent federal legislation, Bill C‐3, was passed by the Government of Canada and received Royal Assent in 2021, which amended the Criminal Code of Canada to, among other things, “create an offense of intimidating a person in order to impede them from obtaining health services, intimidating a health professional in order to impede them in the performance of their duties, or intimidating a person who assists a health professional in order to impede the person in providing that assistance” [15]. While expressing support for this federal legislation to deter violence in health care settings, the CFNU emphasized the need to also develop and implement a “national public awareness campaign to sensitize Canadians on the violence faced by health care workers and highlight the valuable role health care professional play in providing care” [16]. Together, while not shaping the data of this current study, these developments reflect growing recognition of the systematic nature of violence in health care and the need to understand its root causes and manifestations.

The consequences of workplace violence for nurses are well established. Exposure to violence is associated with posttraumatic stress disorder (PTSD), major depressive disorder, generalized anxiety disorder, and panic disorder [1, 17, 18]. In a study of psychiatric staff in Canada, Hilton et al. [19] found that PTSD symptoms were associated with exposure to critical events, including physical threats, assaults, or deaths. Similarly, Olashore et al. [17] examined 201 mental health providers (60.5% nurses) who had been exposed to occupational violence by patients in the previous 12 months and found nurses to be three times more likely to experience PTSD compared to health care providers who did not experience violence. Workplace violence further contributes to decreased motivation, job satisfaction [20], productivity, and quality of care [21], as well as intention to leave the job or profession [22], exacerbating workforce shortages.

While the scientific literature has documented nurses’ experience with violence from many sources (e.g., patients, patients’ families, and colleagues), few studies qualitatively explore the impacts of violence against nurses by patients’ families and other visitors. Quantitatively, the available evidence suggests patients’ families and other visitors may be the second highest perpetrators of violence against nursing staff, following patients themselves [4, 6, 23–25]. For example, one study found more than half of nurses (59%) have reported at least one episode of verbal abuse, and many (20%) have reported up to five episodes of physical violence from visitors in the past year [25]. Recent studies similarly indicate nurses often identify verbal abuse (32.9%) and, to a lesser extent, physical abuse by visitors (3.5%) as their most serious incidents of workplace violence [26].

Despite these findings, qualitative insights into how nurses perceive, interpret, and experience violence from family members and visitors, particularly within the Canadian context, remain limited yet urgently needed given the well‐documented mental health consequences of workplace violence and the ongoing nursing workforce crisis in Canada. In response, the current study examines Canadian nurses’ (*n* = 168) perceptions of PPTEs in the context of violence perpetrated by patients’ families and other visitors. Our findings offer important insights into nurses’ experience with these often‐overlooked sources of violence and provide evidence to inform proactive strategies and policies to improve nurses’ safety and well‐being.

## 2. Materials and Methods

### 2.1. Study Design and Procedures

Our study is part of a larger project examining the prevalence of and experiences with mental health disorders among Canadian nurses. A web‐based self‐report survey was administered to Canadian nurses in French and English through Qualtrics between May and September 2019, before the onset of the global COVID‐19 pandemic. The research team collaborated with the CFNU executive to develop a survey comparable to previous mental health research conducted with Canadian public safety personnel [27], but tailored specifically for nurses. The study design was approved by the University of Regina Institutional Research Ethics Board (File #2019‐052). Prior to any data collection, participants read an online consent form and could only proceed to the survey after providing voluntary informed consent. We did not require any identifying information in the consent process (i.e., we did not collect names or dates of birth). Given data were unidentifiable, we did not have an explicit data de‐identification process.

#### 2.1.1. Participants

The survey was distributed through CFNU members’ email lists and social media pages, unions of non‐CFNU member nurses, as well as nursing associations and colleges in British Columbia and Quebec. Participants completed the survey anonymously through a website link. There were 7358 participants (92.0% women) who started the parent survey, many of whom (*N* = 4067; 94.8% women) completed measures assessing their exposures to PPTE. These participants were then invited to provide additional details on their PPTE exposures in open‐text response fields. The current study focused specifically on participants for whom the worst PPTE involved violence perpetrated by family members or visitors of patients (*n* = 168), which is the final analytical sample for the current study. We do not report information on participants’ work setting or unit, which is not the locus of analysis. Further demographic information on these participants is provided in Table 1.

**TABLE 1:** Participants’ characteristics.

	Participant responses	Percent
*Sample*	168	100

*Gender*		
Male	8	4.8
Female	158	94

*Age group*		
19–29	25	14.9
30–39	33	19.6
40–49	32	19
50–59	34	20.2
60+	5	3
Unassigned	39	23.2

*Job length*		
Under 1 year	2	1.2
1 to under 5 years	23	13.7
5 to under 10 years	40	23.8
10 to under 20 years	37	22
20+ years	66	39.3

*Nursing license*		
Registered psychiatric nurse	8	4.8
Registered nurse	137	81.5
Nurse practitioner	1	0.6
Licensed practical nurse/registered practical nurse	21	12.5
Other	1	0.6

### 2.2. Measures

In the broader survey, lifetime PPTE exposures were measured with the Life Events Checklist 5 (LEC‐5) [28]. For details on how the LEC‐5 was used and revised within the current survey, see Stelnicki et al. [29]. The revised LEC‐5 consisted of the original 17 items with an additional five nursing‐specific exposure items (i.e., death of a child, death of an individual after extraordinary efforts to save their life, death of someone reminiscent of a friend or family member, exposure to a life‐threatening epidemic, and all other events). In addition to being asked the standard LEC items, participants were asked to select the exposure that was the worst or most impactful for them (i.e., the PPTE specific to their nursing career with the most significant lasting implications for their mental health and well‐being) and complete an optional open text box to enter any comments about the event. There were 14 questions assessing nursing‐specific PPTEs, and an “other PPTE” unlimited character open‐text response option. Data collected from responses to the “other PPTE” option were included in the current analysis; the prompt read: “Have you had any experiences that are not listed above?” At the end of the PPTE exposures section of the survey, participants were asked “do you have any other comments regarding these events?” (i.e., “other comments”). Data provided in the “other comments” open‐text response field were also included in the current analysis. These open‐ended responses were coded and categorized. Individual responses ranged from a sentence to paragraphs. Participants who reported violence perpetrated by a family member or other visitor were included in this analysis.

### 2.3. Analysis

We primarily engaged a realist orientation, which guided our approach to qualitative data as constituting a reality or truth whereby concerns for objectivity could be addressed and mitigated, irrespective of the limitations of generalizability that come with most forms of qualitative research [30]. Responses in the open‐text response fields were analyzed using a semigrounded approach [31, 32], or more specifically, grounded theory–informed thematic analysis. Most forms of thematic analysis are epistemologically flexible [33]; as such, we oscillate in this study analytically from realist to grounded to (somewhat) constructivist positioning of experiences shared, a positionality helping us to navigate complex realities and interpretations of violence. This meant themes were produced from these data, though we did not suspend our theoretical knowledge during the analytical process [34]. However, new theory did not emerge from these data, as described with grounded theory; rather, we applied and built on existing theory to explain the themes and related nuance. The coding team consisted of Master’s and PhD level researchers who each coded diverse sections of these data, with the support of a coding manger, and met regularly to discuss any discrepancies in how data were coded. Consensus was always reached through discussion and constructive disagreement until resolution. Consistent with the aims of this study, data were approached as a whole dataset, rather than subsets based on demographic factors, as visitor violence was not localized to specific settings or locations. Further, French data were translated by a multilingual French research assistant and coded within the greater dataset.

In reading through these data, we identified the overarching themes or larger categories of discussion, and then the team deconstructed the themes into unique codes to nuance the different ways each appeared and was articulated by participants. Codes were then analyzed across participants and within the participants’ individual responses. This process included using NVivo software to open code these data into parent nodes (the theme) and child nodes/grandchild nodes (i.e., nuanced elements of the themes). After all data were coded, we merged the coded themes from the two items because of their overlap (i.e., other nursing exposures and additional comments on exposures). Codes were then analyzed across participants and within the participants’ individual responses with a focus on how nurses experienced the violence perpetrated by patients’ family or other visitors. Rigor was maintained throughout the analysis through double‐coding of data, weekly team discussions on coding categorizations, and peer consults with nurse colleagues.

## 3. Results

Participating nurses (*n* = 168) described experiencing violence from patients’ family or other visitors as being the worst PPTE endured, leading (in their view) to the greatest, lasting implications for their mental health and well‐being. In the following analysis, we focus on qualitative themes emerging from open‐ended survey comments related to this phenomenon, specifically normalization of harm and violence, verbal abuse, physical violence, and emotional abuse. We summarize these themes in Table 2. Frequency counts for each theme were intentionally excluded, as the occurrence of interactions may not reflect their significance or impact, and the study focused on the qualitative nature of these experiences rather than their prevalence.

**TABLE 2:** Summary of themes.

Theme	Description	Exemplar quote with participant ID
Normalization of harm and violence	Nurses perceptions of violence as normalized and expected, reflecting on organizational culture that accepted violence as part of the job	“Accepted as part of the job” (P1960)
Verbal	Nurses descriptions of verbal violence by patients’ families or other visitors	“Manipulated, lied to, (and) berated by family members” (P1046)
Physical	Nurses descriptions of physical aggression toward them by patients’ families or other visitors	“A patient’s partner physically attacked me in a hallway when I was a new graduate” (P2545)
Emotional	Nurses descriptions of types of emotional or psychological violence	“It is extremely demoralizing to be talked to that way” (P1607)

### 3.1. Normalization of Harm and Violence

Violence from patients’ family and other visitors was described by nurses as occurring in several different work settings. For example, P49 explains:

I have worked in several PCHs and transitional care units, and verbal abuse and aggression from clients and/or client family members is something that has been present at every site (P49).

Problematically, participants reported that poor treatment from patient families and other visitors is “accepted as part of the job” (P1960) and, given the time of the current study, participants did not benefit from legislative changes to deter health care violence that occurred after survey participation; thus, participants felt there was no recourse for such behaviors. To elaborate, nurses must regularly manage distressed family members, whose behaviors can be inappropriate, unkind, and even criminal. Participants implied that the absence of meaningful and proportionate recourse and consequences was a critical organizational driver for the ongoing abuses and left nurses feeling without options in such situations. For example, P744 explains:

Patient’s family members yelling and cursing at staff. In all of these events when reported our managers say we are to smile and keep the peace and not report anything because there is nothing they can do. We wouldn’t want to have a negative perception in the public eye (P744).

Here, P744 raises the concern of liability in relation to public perception. Specifically, if nurses were to speak out or resist health care violence, members of the public might vilify or further aggress against staff. With cultural “acceptance” of such violence becoming normalized and claims circulating that little, if anything, can be done to curb the violence, P1960 felt silenced and described how their own physical safety was irrelevant in the opinions of other staff.

… physical/verbal abuse by patient/family is expected to be ’dealt with as a professional’ by just getting on with work. There is no recourse to the attacker, it is just accepted as part of the job, no matter the injury. Something that is rarely spoken of, and never dealt with in a legal way, i.e., banning abusive visitors or charging the abuser. We are told that charges will never be laid nor stick (P1960).

When violence occurs against nurses, discourses that warn against or are cautious toward the pursuit of justice are thought to prevail culturally, thereby reinforcing mechanisms of normalization and/or blame. In P1960s account, these discourses contributed to a silencing effect in which violence was minimized or did not result in meaningful consequences. Nurses who were victims of violence described not only the violence itself but also the secondary harm of feeling blamed for the incident.

This is what it is to be a nurse. We have patients and families often who are rude, occasionally threatening. Majority of the time we are made to feel we did something wrong and these people get away with it (P1106).

P1106 reported feeling she “did something wrong” and having no recourse against the violent family members. P1351 similarly reported “blaming health care staff” as common, irrespective of systematic challenges that impede patient health care. These narratives signal an important challenge to Canadian nurses who feel compromised in terms of their safety, yet are made to believe the onus of responsibility for preventing or responding “appropriately” to such behaviors is on the individual nurse, rather than the organization and its leaders. In this way, organizational responses characterized by minimization, blame, and a lack of clear consequences contributed, in participants’ views, to the normalization of violence. Thus, these accounts suggest that inadequate organizational and leadership responses to violence represent to participants a compounding and secondary harm to nurses.

#### 3.1.1. Verbal Abuse

Participants reported experiencing pervasive and enduring verbal abuse and threats from patients’ families or other visitors, with some describing such exposures as the worst PPTE experienced. For example, patients’ family members were described by nurses as “a major cause of stress” (P1046), with nurses being “manipulated, lied to, (and) berated by family members” (P1046). Another participant echoed “The family members are equally if not more abusive than my patients” (P2178). P48 and P417 shared further and disturbing evidence of traumatic exposure to violence:

The friends and families of patients can also be very verbally abusive and have threatened bodily harm to staff. I once had a man tell me he would be waiting for me in the parking lot after work. To take care of me. I was terrified for several weeks when I had to arrive or leave the hospital (P48).

…family member of one of our patients verbally abused and threatened all of our staff and doctors, threatening to bring a gun to the facility. Causing stress and anxiety amongst the staff during the continuation of her treatment (P417).

Both participants describe long‐term fear responses to violent threats by patients’ family members or other visitors as their worst PPTE at work, and neither participant reports how the organization responded to these threats, except to say how staff managed the problem individually while still being obligated to attend work and continue treatment for the patient during clear duress. Racialized or sexually inappropriate comments made by patients’ family or other visitors were also reported by nurses: “Insensitive racial comments against my ethnicity from patients and family of patients. Inappropriate sexual comments about my body…. Racist and sexist comments against nurses from patients and their families affect my self‐confidence and my ability to fully care for them” (P1768). Another participant wrote, “One more thing, as (an) African nurse I experience racist behaviors consistently from patients/families and at times from other health care workers without anyone to recognize it or even stop it” (P4655). What is again evident is how participants believe these problems are not dealt with meaningfully by peers or through organizational mechanisms, creating heightened and lingering feelings of stress, low self‐esteem, and in turn degrading the levels of care nurses can provide in unsafe and toxic work environments.

Participants reported other PPTEs, including threats from patients’ families to report, pursue legal action, or encourage an investigation against them:

…patient families‐verbal abuse, threats to report to the CNO, complaints about patient care that are unwarranted ‐ there is more and more pressure to keep the family happy even if it’s not necessarily in the patient’s best interest in the long term (P540).

Here, P540 describes being ensnared by concerns for liability to the point they become willing to alter care and their approach to the patient (i.e., “keep the family happy”), even if that means the patient’s best interests and care goals in the long term may not be realized.

#### 3.1.2. Physical Violence

Participants also described experiences of physical violence enacted by family members of patients. For example, one nurse wrote:

Patients & their families don’t cope and they escalate on nurses. I have experienced verbal aggression with physical gestures of violence to hospital equipment often (P2359).

Violence directed at staff was a particularly distressing PPTE that appeared to generate long‐term negative impacts on nurses’ mental health and well‐being:

A patient’s partner physically attacked me in a hallway when I was a new graduate. Police were called. He was arrested. Since then, I have problem with personal space (P2545).

… a family member who I was trying to help by going to get a buzzer pinned me against a wall. I blacked out for a few seconds and cried profusely but went right back to work. No nurse wants to be charged with abandoning their patient… (P3537)

The excerpts highlight patients’ family members attacking participants, and due to the silencing effect around consequences and pursuits of justice, P3537 became concerned after being attacked about the liability around “abandoning” their patient. It is clear how participants often frame the patients’ interests as trumping the safety interests of staff, and whether or not nurses agree with this positionality, most seem to carry an extra burden that is creating oft‐unaddressed stress within their organizations. Importantly, P2545’s experience of physical assault ended with the police intervening, but not without lasting impacts; overall, participants collectively seem to experience violence at work, limited support and recourse, and negative mental health sequelae.

#### 3.1.3. Emotional Abuse

Participants described experiences of emotionally abusive family members and other visitors as their most impactful PPTE exposure. Beyond the imposition of blame, nurses spoke of “emotional abuse by patient’s families” (P647) and being “emotionally … attacked” by families (P1016). For example, participants wrote:

Family was present and blaming health care staff. Prolonged wait times contributed to the situation (P1351).

Even though there is no truth/evidence to the verbal threats from anti‐vax parents, it is extremely demoralizing to be talked to that way (P1607).

P1607 describes how differences in health care beliefs between nurses and patients, in this example, vaccinations, can create inescapable negative emotional workplaces for nurses. The differences can impede nurses’ ability to provide effective patient care, create hostile work environments, and harm nurses’ mental health. For circumstances largely out of the control of nursing staff, such as wait times, these predicaments can drain nurses and raise concerns about the emotional labor of the occupation, worsened by violence and emotional abuse.

## 4. Discussion

Our study provides qualitative evidence for how the violence perpetrated by family and other visitors constitutes nurses’ most impactful PPTEs across diverse settings in Canada. Furthermore, we highlight the pervasive organizational normalization and underreporting of these incidents by nurses. In the study, many nurses described experiencing a range of verbal, physical, and emotional violence/abuse from family members and visitors as their most impactful PPTE, advancing the idea that violence from family members and visitors continues to have a largely negative impact on nurses’ mental health, well‐being, and sense of belonging in their occupation [1, 4, 5]. Notably, in the present study, these types of violence (i.e., verbal, physical, and emotional) from family members and other visitors were found across all types of care settings, which is consistent with previous findings of violence as a widespread phenomenon in health care settings, and not confined to any particular setting (i.e., emergency room), as previously thought in early research [5].

Nurses reported that the frequency and pervasive nature of violence against nurses contributes to the normalization of violence as “part of the job.” Normalization broadly refers to the social processes wherein ideas, perceptions, and norms become taken‐for‐granted in everyday life and culture and which are actively shaped standards for moral evaluation in each group [35]. Many of the events described by nurses involved risk of serious physical harm or psychological injury that would normally be chargeable but are instead accepted as inherent to the nursing profession. Nurses in this study reported that normalization was often reinforced by organizational and leadership responses interpreted as implicitly or explicitly attributing violent incidents to perceived professional or interpersonal failings of nurses, thereby diverting attention away from the structural conditions enabling violence.

Previous research has similarly tended to frame violence as stemming from how nurses provide care, suggesting violence may occur when family members and other visitors become frustrated over their care, or perceived lack of care provision [6], or when there are communication failures between nursing staff and family members [25]. Such explanations, however, risk placing responsibility for violence on individual nurses and obscuring broader organizational and structural contributors. For example, Nabb [25] and others [8, 9] show how multiple systemic issues affect care, like how low staffing levels make spending time talking to family, increased workload demands, and higher levels of care expectations than can realistically be provided all difficult. Findings from the current study reinforce the importance of similar organizational stresses. Participants described leadership responses that attribute violence incidents to nurses’ alleged professional shortcomings, which nurses interpreted as prioritizing the needs of patients’ family members or other visitors over nurses’ safety. These responses align with emerging evidence linking workplace violence to moral distress and harm, and in some cases, moral injury when nurses and other health care providers are left feeling unsupported or betrayed by their leadership and organization [36–38]. Similarly, Lyneham [24] noted leaders are more likely to address violence against physicians rather than nurses, further amplifying perceived inequities. Leadership inaction, inconsistent policy enforcement, or blaming nurses for violence may exacerbate the process by eroding trust and reinforcing perceptions of organizational abandonment. This is consistent with evidence suggesting that a lack of leadership support further intensifies feelings of victimization and traumatization [39]. Further research is needed to explore the links between workplace violence and moral distress/injury and how leadership responses can mediate or reduce these harmful outcomes.

Our results also demonstrate how shifting the responsibility for the prevention and management of violence from the organization to the nurse has additional consequences. Examples included how participants were hesitant to document these incidents due to concerns about reprisals or lack of managerial follow‐up. Notably, the current results are still consistent with previous research estimating that only 19% of violent incidents against nurses are reported [24], further emphasizing the magnitude of the problem, which may be larger than previously understood. This apparent “silencing” of violence may be related to the perception that reporting will not yield any substantial change or decrease in violence [26]. Greater support by management has been suggested to prevent workplace violence [26]; however, other studies suggested front‐line nurse managers lacked the necessary financial and organizational resources to effectively prevent and manage violence against nurses, leaving only reactive response options such as providing access to counselors [40]. Having insufficient resources and only reactive response options inevitably limits the capacity of nurse leaders to address the underlying and systemic factors facilitating violence against nurses.

During the COVID‐19 pandemic, nurses reported an increase in the frequency and intensity of physical and verbal–emotional–psychological workplace violence, primarily perpetrated by patients’ family members [41]. The cumulative consequences of increasing workplace violence against nurses are taking a toll and are likely to have substantial negative impacts on retention [42]. For example, O’Connell et al. [5] found that after a workplace aggression incident, 20% of nurses take sick leave, 20% take alcohol or drugs, and 8.6% change units or employers [5]. Another study found nurses frequently described fear of returning to work after violent incidents and, in some cases, considered leaving their positions [25]. Among those who remain, burnout is common; more than half of nurses report feeling “burnt out” following workplace aggression [5].

Although our study was conducted prior to the pandemic, participants’ descriptions align with broader evidence documenting how violence from family members and visitors remains an ongoing and potentially psychologically traumatic aspect of nursing work, with serious implications for their mental health and well‐being.

Given that workplace violence is consistently identified as one of the most anxiety‐provoking aspects of nursing practice [26], and is closely related to high rates of burnout (63%) and comorbidity with mental health disorders among Canadian nurses [1], addressing workplace violence is urgently required as health care systems struggle (d) to maintain their nursing workforce throughout the challenges of COVID‐19 and beyond [29, 41, 42]. Although rates of violence against nurses have always been problematic, the frequency has dramatically increased alongside exacerbated mental health challenges during the COVID‐19 pandemic, increasing the likelihood that many nurses will leave the profession and many more will never enter [29, 41]. Urgently addressing increasing levels of violence against nursing staff is a responsibility of organizations and leaders who want to protect the mental health of nurses [43], which requires addressing violence to perhaps help with the growing problems of retention and recruitment [42].

An important step in addressing workplace violence would be formal and public policies of zero tolerance for violence against nursing staff. In Canada, Royal Assent was granted to Bill C‐3 in December 2021, which amends the Criminal Code of Canada by making it an offense to intimidate a health professional in the performance of their duties [15]. Importantly, organizational antiviolence policies that include enforcement and appropriate sanctions for transgressing family members and other visitors may offer important initial opportunities for improvements [4, 7], but they are not sufficient on their own. Preventing and reducing violence is a complex problem that requires a multifaceted approach. Such change must begin with a culture shift in health care organizations that prioritizes the safety and well‐being of all staff, including nurses.

Another important step involves nursing leaders actively enforcing antiviolence policies and sanctioning persons who transgress the policies [4, 44]. Leadership plays a pivotal role in shaping workplace culture. When leaders demonstrate a commitment to staff safety through actions, such as endorsing zero‐tolerance policies, allocating resources for security, and using incident reports to focus on systemic concerns, rather than individual factors, leaders help to reinforce trust by clearly signaling to nurses that violence will not be dismissed as “just part of the job.” Studies suggest proactive leadership, increased presence, and adequate support are among the more effective institutional strategies to prevent and respond to workplace violence [12, 45, 46]. Organizational structures are therefore necessary to support leaders in operationalizing antiviolence strategies through established levers, including established visitor management protocols and timely security escalation pathways that are consistently enacted following incidents of violence. In addition, implementing transparent accountability mechanisms that acknowledge the structural and systematic contributions to violence, rather than placing responsibility solely on individual nurses, may help improve reporting practices and reduce reporting futility. Such mechanisms are also important for identifying recurring sources and patterns of violence, thereby informing more effective organizational prevention and response strategies.

Health care organizations must also implement broader strategies aimed at preventing workplace violence, including educating staff and leaders, creating safer work environments, and introducing additional types of protective measures where appropriate, such as portable alarms [4, 7]. Staff may also become more confident in dealing with aggressive behavior after attending in‐service education focused on managing violence [47].

Furthermore, adopting a just culture approach to incident response and organizational accountability may be necessary [48]. In a just culture, incidents are treated as opportunities to examine and correct systemic failures rather than to assign blame to individual staff members, which fosters psychological safety and encourages staff to report incidents of violence without fear of retaliation or stigma. By contrast, a blame‐oriented culture suppresses reporting, conceals the true magnitude of workplace violence, and perpetuates the normalization of abuse [48]. Our study’s findings, where nurses interpreted leadership blame as prioritizing visitor comfort over staff safety, underscore why a shift toward just culture is essential.

Organizations and leaders must ensure that nurses receive timely and meaningful support following incidents of violence. Such support should extend beyond immediate incident management and include access to emotional and psychological supports, transparent communication regarding actions, and accommodations when needed. Leadership visibility and engagement following incidents may also be important in reinforcing that nurses’ safety concerns are taken seriously and that violence is not tolerated in the workplace. Collectively, these supports may help mitigate the longer‐term emotional impacts of workplace violence, strengthen trust in leaders, and encourage future reporting.

Finally, an equity‐oriented lens is also necessary when addressing workplace violence. Participants’ accounts of racism, sexism, and other discriminatory behaviors suggest that workplace violence may be compounded by broader systems of inequality in health care environments and society. Organizational strategies must also include targeted antidiscrimination policies and measures to ensure that all nurses are protected and supported.

### 4.1. Limitations

The study has several important limitations. First, the analysis draws on self‐selected participants who provided brief open‐ended responses, which restricts interpretive depth. Since these were written comments rather than interview data, we could not probe, clarify meaning, or explore contextual details, and these data reflect what participants chose to disclose about their experience. As a secondary analysis, the findings capture broad patterns but cannot provide the same nuance as in‐depth interviews. Anonymity may also both enable disclosure and reduce contextual detail (setting, role, and perpetrator specifics). In addition, a substantial number of participants chose not to provide demographic data, limiting interpretation. Accordingly, future interview‐based research is required that also controls for experience of visitor violence as tied to factors such as demographics (i.e., age) and area of work (e.g., long‐term care, psychiatric units). Second, although the sample for the current study was largely members of the CFNU, representing almost 200,000 nurses and nursing students across eight Canadian provinces, the sample may not be entirely representative of the nursing workforce. That said, the sample demographics were generally proportionally consistent with the age and gender of Canadian nurses. However, nurses from all provinces are not represented, and the voluntary nature of their participation introduces potential self‐selection and recall biases. Moreover, because responses were anonymous and not linked to workplace characteristics, we lacked setting‐level denominators needed to assess for differential risk across settings. The inability to link responses to organizational characteristics (e.g., setting) limits conclusions about which management interventions are most feasible. Third, there was no opportunity for triangulation with other data sources, such as incident reports or administrative records, nor could we conduct member checking. Finally, while the survey guaranteed anonymity, social desirability possibly may have shaped under‐disclosure of experiences. Furthermore, these data for the current study were collected in 2019, and subsequent research suggests nurses have experienced substantially increased stress since COVID‐19 [29, 49]; accordingly, the current results likely underrepresent the size, scope, and severity of the challenges nurses face.

### 4.2. Implications for Nursing Management

These findings support the need for system‐level organizational responses that prioritize nurse safety and well‐being. Priority actions for health care organizations and leaders could include (1) strengthening accountability for workplace violence prevention and response by supporting leaders with clear protocols and resources; (2) implementing and consistently reinforcing clear organizational policies; (3) fostering a just culture approach that emphasizes system learning and improving rather than assigning blame; (4) improving and encouraging timely documentation and response; and (5) enhancing training and supports for staff in recognizing, de‐escalating, and responding to violent incidents from family and other visitors. Collectively, these strategies underscore the importance of coordinated, multilevel approaches rather than isolated interventions. Addressing workplace violence in a systematic way is essential to supporting nurse retention and a strong, healthier nursing workforce in Canada.

## 5. Conclusion

We are facing a global shortage of nurses [50, 51]. Now more than ever, we need strategies to recruit and retain nurses in the profession. Despite repeated calls for addressing violence against nursing staff, violence continues to be a major problem affecting nurse retention and physical, mental, and emotional safety. Our findings underscore how violence from family members and visitors remains a persistent issue threatening nurses’ physical and psychological safety. Researchers must continue to explore and unpack the organizational contexts and strategies needed to address all forms of violence against any health care worker, and more importantly, work with organizations to action such strategies in the here and now.

## Author Contributions

Kim Ritchie: writing–original draft, reviewing, and editing. Rosemary Ricciardelli: article conceptualization, qualitative methodology, formal analysis, writing–original draft and editing, and supervision. Matthew S. Johnston, Andrea D’Alessandro‐Lowe, Margaret McKinnon, and Yuanxin Xue: writing–reviewing and editing. Andrea M. Stelnicki: data curation, reviewing, and editing. R. Nicholas Carleton: study conceptualization, study methodology, funding acquisition, and reviewing and editing.

## Funding

The authors acknowledge the financial support from the Canadian Federation of Nurses Unions (CFNU) that made the current research possible. Specifically, Andrea M. Stelnicki received financial support for the current research in the form of postdoctoral funding from CFNU.

## Conflicts of Interest

The authors declare no conflicts of interest.

## Data Availability

The data that support the findings of this study are available on request from the corresponding author. The data are not publicly available due to privacy or ethical restrictions.
